# Formulation Strategies of Nanosuspensions for Various Administration Routes

**DOI:** 10.3390/pharmaceutics15051520

**Published:** 2023-05-17

**Authors:** Sıla Gülbağ Pınar, Ayşe Nur Oktay, Alptuğ Eren Karaküçük, Nevin Çelebi

**Affiliations:** 1 Department of Pharmaceutical Technology, Faculty of Pharmacy, Süleyman Demirel University, Isparta 32260, Turkey; silagulbag@sdu.edu.tr; 2 Department of Pharmaceutical Technology, Gülhane Faculty of Pharmacy, University of Health Sciences, Ankara 06018, Turkey; aysenur.oktay@sbu.edu.tr; 3 Department of Pharmaceutical Technology, Faculty of Pharmacy, Ankara Medipol University, Ankara 06050, Turkey; alptug.karakucuk@ankaramedipol.edu.tr; 4 Department of Pharmaceutical Technology, Faculty of Pharmacy, Başkent University, Ankara 06790, Turkey

**Keywords:** nanosuspensions, formulation strategies, production methods, administration routes, dosage forms

## Abstract

Nanosuspensions (NSs), which are nanosized colloidal particle systems, have recently become one of the most interesting substances in nanopharmaceuticals. NSs have high commercial potential because they provide the enhanced solubility and dissolution of low-water-soluble drugs by means of their small particle sizes and large surface areas. In addition, they can alter the pharmacokinetics of the drug and, thus, improve its efficacy and safety. These advantages can be used to enhance the bioavailability of poorly soluble drugs in oral, dermal, parenteral, pulmonary, ocular, or nasal routes for systemic or local effects. Although NSs often consist mainly of pure drugs in aqueous media, they can also contain stabilizers, organic solvents, surfactants, co-surfactants, cryoprotectants, osmogents, and other components. The selection of stabilizer types, such as surfactants or/and polymers, and their ratio are the most critical factors in NS formulations. NSs can be prepared both with top-down methods (wet milling, dry milling, high-pressure homogenization, and co-grinding) and with bottom-up methods (anti-solvent precipitation, liquid emulsion, and sono-precipitation) by research laboratories and pharmaceutical professionals. Nowadays, techniques combining these two technologies are also frequently encountered. NSs can be presented to patients in liquid dosage forms, or post-production processes (freeze drying, spray drying, or spray freezing) can also be applied to transform the liquid state into the solid state for the preparation of different dosage forms such as powders, pellets, tablets, capsules, films, or gels. Thus, in the development of NS formulations, the components/amounts, preparation methods, process parameters/levels, administration routes, and dosage forms must be defined. Moreover, those factors that are the most effective for the intended use should be determined and optimized. This review discusses the effect of the formulation and process parameters on the properties of NSs and highlights the recent advances, novel strategies, and practical considerations relevant to the application of NSs to various administration routes.

## 1. Introduction

In recent years, one of the most challenging issues encountered in both the pharmaceutical industry and pharmaceutical studies has been drug candidates with low water solubility [1]. The main challenges are that the dose-response linearity of drugs with low water solubility may decrease, and unexpected collapse of the drug may be encountered after administration, leading to decreased patient compliance and decreased bioavailability. In addition, due to the low solubility of active substances in water, variations may occur as a result of changing the absorption of the drug in fasted and fed states [2]. There have been very promising developments in the studies carried out in the last century to overcome these problems, and the most important of these is the development of nanosized drug delivery systems. The basis of these approaches is related to the increase in solubility when surface area is increased, depending on the Noyes–Whitney equation as a result of reducing the particle size of the active substance, thus increasing the dissolution and bioavailability [3]. To increase solubility and thus bioavailability, drug delivery systems such as liposomes [4], nanoparticles [5], solid lipid nanoparticles [6], polymeric micelles [7], dendrimers [8], quantum dots [9], nanoemulsions [10], and nanosuspensions [11,12] are the most widely used. Many studies on nanosuspensions using poorly soluble drugs have been conducted since nanosuspensions were first reported by Müller et al. in 1994 [13].

Nanosuspensions (NSs) are colloidal dispersions of submicron drug particles and are generally defined as very finely dispersed and biphasic colloids containing solid drug particles smaller than 1 μm [14]. Although there are some differences in the literature on the definition of nanosuspensions, and the words *nanosuspensions* and *nanocrystals* are used interchangeably in these studies, they are characterized as “pure active pharmaceutical ingredients (APIs) between 10–1000 nm stabilized with surfactant or polymer” [15], as well as “particles with a particle size of approximately 200–600 nm below 1 micrometer, formed by 100% pure active substance” [16]. An NS is expressed when prepared with stabilizers in the form of nanosized drug crystals.

NSs have many advantages over other drug delivery systems. These advantages can be summarized as follows:

- NSs provide enhanced oral bioavailability of drugs by increasing the saturation solubility and dissolution of the active substance and by increasing adhesion to the cell surface membranes [17].
- NSs can also allow passive targeting because the particle is of nanometer size [18].
- They are simple, easy, and inexpensive to produce, and they themselves produce rapid and reproducible formulations [19].
- Production costs are very low because of the low excipient requirements during their preparation. Moreover, their production can be scaled up [20].
- They reduce the bioavailability differences in fasted/fed states caused by the effects of food [21].
- They reduce inter-subject variability in bioavailability [17].
- They have a high drug content (accepted as 100%), so the dose used is reduced in therapy [22].
- Physical stability is increased in solidified nanosuspensions, and solidified formulations can be presented to patients in solid dosage forms such as tablets or capsules [17].
- NSs can be formulated for parenteral, pulmonary, topical, and ophthalmic routes of administration, in addition to the oral route [14].
- They can be sterilized by various methods such as filtration, dry heat, steam, and radiation [23].

The many advantages of NSs (or nanocrystals) have led to the development of many commercial products produced with nanocrystal technology in the pharmaceutical industry. Nanocrystal-based formulations are widely used to treat cancers, pains, nausea, asthma, hypertension, hypercholesterol, inflammatory diseases, cardiovascular diseases, bronchial dilatation, depression, dermal diseases, and other diseases [24,25]. There are many clinical trials related to NSs in different phases (such as Phase II and III) [26].

In spite of these many advantages and numerous commercially available products, NSs also have several disadvantages:

- The formulation may not be suitable for some pharmaceutical active ingredients, and difficulties may be encountered in choosing the stabilizer type and stabilizer ratio used in the formulation.
- There is a potential for physical stability problems in liquid form during preparation in nanosuspension form [19].
- Particle growth may occur in the drying step because of insufficient cryoprotectant power [27].
- Undesirable polymorphic changes may be encountered because of the need to use devices (high-pressure homogenizers or wet bead mills) in the preparation of NSs and because of the high pressure and temperature increase and of the mechanical power applied accordingly [19].

In the first part of this review, an overview of the preparation methods of NSs, stabilizers used, characterization studies, and solidification techniques will be given. The second part addresses the routes of administration of nanosuspensions for systemic or local effects, and each route of administration is summarized in tables.

## 2. Preparation Methods for Nanosuspensions

**Figure 1:**
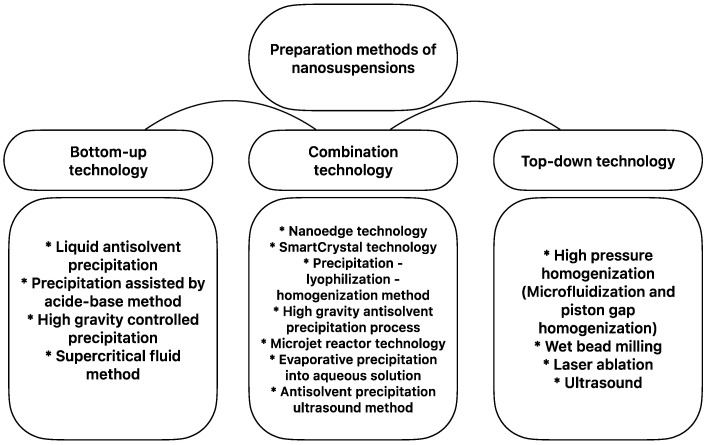
Nanosuspension preparation methods (conventional and combination technologies).

Many methods have been developed by research laboratories and pharmaceutical experts for the preparation of NSs, and these methods are broadly divided into three categories: bottom-up technology, top-down technology, and a combination of these two (Figure 1). Apart from these methods, other preparation techniques, such as supercritical fluid technology, an emulsification–solvent evaporation method, and a melt emulsification method, have also been successfully developed in line with advanced studies [24].

### 2.1. Bottom-Up Technology

**Figure 2:**
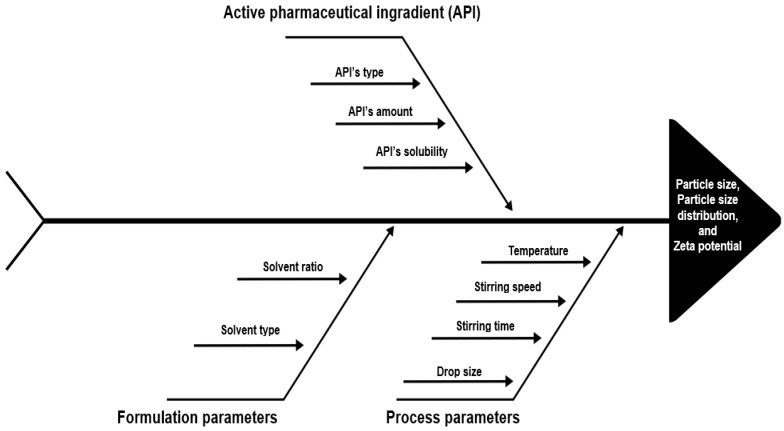
Schematic representation of the critical parameters of bottom-up technology by the fishbone diagram.

The bottom-up technology, which is also referred to as “nanoprecipitation”, was first used in 1987 by List and Sucker [28]. This bottom-up technology is based on the principle of obtaining nanosized particles by precipitating dissolved molecules with the addition of another insoluble substance. For this method to be applicable, the active substance must be soluble in at least one solvent and suitable stabilizers must be used to prevent the growth of particles after precipitation [17]. In Figure 2, the parameters that affect particle size and particle size distribution in NS formulations obtained by the bottom-up method are shown by the fishbone diagram. The advantages and limitations of the bottom-up method are summarized in Table 1.

Newly developed bottom-up methods such as liquid antisolvent precipitation (LAS), precipitation assisted by the acid-base method, high-gravity-controlled precipitation (HGCP), the supercritical fluid method (SCF), and the emulsion polymerization method are also available in the literature [24].

### 2.2. Top-Down Technology

Top-down technologies are based on the reduction of large particles down to the nanoscale. The main methods used include the high-energy process called high-pressure homogenization and the low-energy process called media milling [14]. These methods are more suitable for industrial production than bottom-up technology, and they are applied to currently marketed products [16].

#### 2.2.1. High-Pressure Homogenization Method

**Figure 3:**
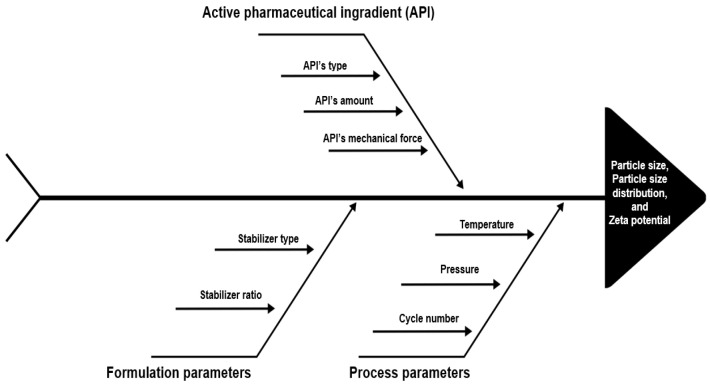
Schematic representation of the critical parameters of the high-pressure homogenization method by the fishbone diagram.

The high-pressure homogenization (HPH) method relies on excessive shear forces and possibly cavitation, which is performed by pressing a suspension from voids or crevices and applying it to the drug crystals to disperse them. The two homogenization principles applied and the type of homogenizer used in line with these principles are microfluidization and piston-gap homogenization. Microfluidization is based on a jet-stream principle in which the coarse suspension accelerates and passes through the homogenizing chamber, especially under the influence of high-speed collision, shear, and cavitation forces, and the particle size becomes smaller as a result of these forces [29]. There are two types of chambers used in this method, the “Z” type and the “Y” type. When in the “Z”-type chamber, the suspension changes several times in the direction of flow, causing particle collision and shear forces; in the “Y” type, the suspension current is split into two streams, which then collide from the front [16]. In the second homogenizer type, the piston gap homogenizer, the coarse suspension passes through a very fine gap at an extremely high speed. The pressures applied in all these processes can vary from 500 bar to 350 Mpa [30,31]. Increasing the pressure and number of cycles generally allows for the preparation of NSs with smaller particles [32,33]. In Figure 3, the parameters that affect particle size and distribution in nanosuspension formulations obtained by the high-pressure homogenization method are shown by the fishbone diagram. The advantages and limitations of this method are summarized in Table 1.

#### 2.2.2. Wet Media Milling Method

**Figure 4:**
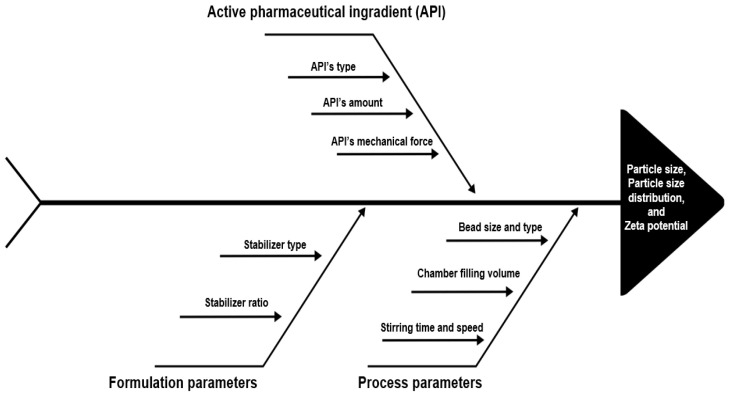
Schematic representation of the critical parameters of the wet milling method by the fishbone diagram.

The media milling method was discovered by Liversidge et al. in 1992 [34]. The most widely used is ball mills, which are used in the preparation of nanosuspensions by a grinding method, although jet mills or colloid mills are also used. The ball mill method can be expressed in different ways: bead milling, wet media milling, and pearl milling. In this method, the substance and stabilizer solution are put into a chamber and mechanical grinding is achieved with the help of balls (beads) hitting it [1]. The wet media milling method involves a milling chamber, milling beads, a suitable stabilizer, and a dispersion medium, usually distilled water. The active substance is dispersed in this dispersion medium, and this coarse suspension is added to the milling chamber [35]. An average of one-third of the chamber is filled with dispersion medium and one-third with milling beads; the remaining one-third is left empty to provide the necessary space for milling [36]. Beads (zirconium, stainless steel, etc.) that are suitable for the process, of the desired number (amount of beads in mL or weight), and size (different bead diameter) are added to this chamber; the rotation speed of the device is adjusted, and the milling process begins with the milling time. The most common problem in this method is the wear caused by the milling chamber or the impact of the beads. It is necessary to use a chamber made of a material such as stainless steel or porcelain and beads made of porcelain, glass, agate, zirconium oxide, or chrome [37]. In Figure 4, the parameters that affect particle size and distribution in NS formulations obtained by the wet milling method are shown by the fishbone diagram. The advantages and limitations of the wet milling method are summarized in Table 1.

**Figure 5:**
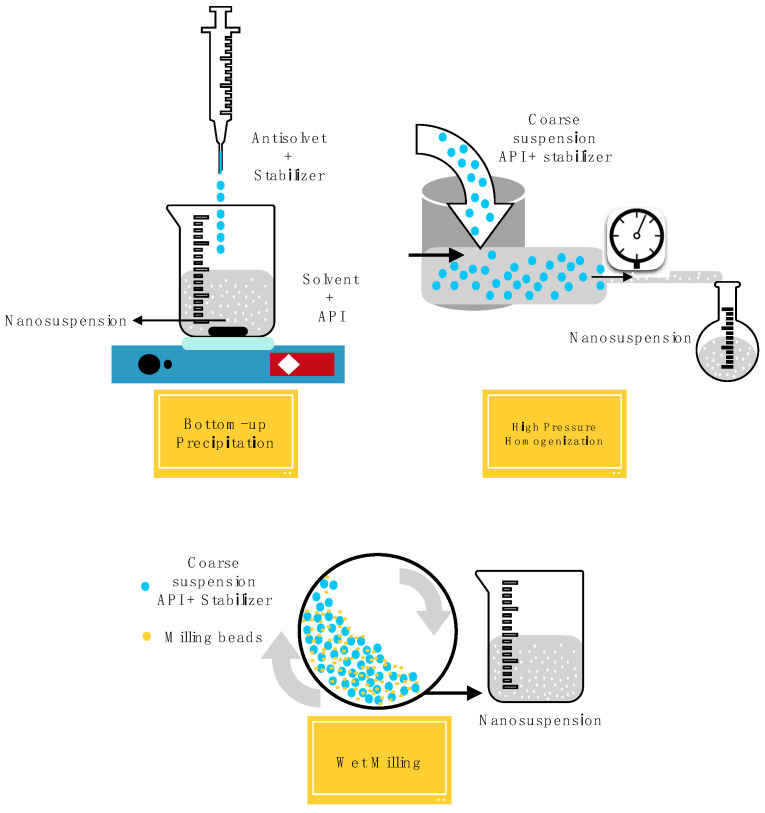
Schematic representation of the nanosuspension preparation methods.

A schematic representation of all the preparation methods described above is summarized in Figure 5.

### 2.3. Combination Technology

In addition to these two technologies (bottom-up and top-down), it is possible to use several techniques together in the preparation of NSs and to obtain NSs with desired properties by making some modifications [20,38]. There are studies in the literature regarding the use of more than one method in combination in Table 1, and these studies also provide an evaluation of the advantages of the above-mentioned methods. With combined methods, it is possible to prepare NSs of the obtained formulation using bottom-up technology and then top-down technology or vice versa.

## 3. Selection of Stabilizers

Stability is crucial for NSs as with all other drug delivery systems. During the preparation of NSs, problems such as attraction or agglomeration may be encountered as a result of the reduction in size of the particles [39]. Because the particles are small and have high energy, it is usual for the particles to grow because of recrystallization or the Ostwald ripening effect [40]. Stabilizers are used in formulations to prevent the particle growth that causes instability in NSs [12,41].

The development of successful NSs is mainly based on the selection of suitable stabilizers. Several stabilizers such as surfactants or polymeric excipients were evaluated for the optimization of the NS. Parameters of stabilizers such as type, ratio, and molecular weight must be evaluated for the stability of prepared NS. In addition, the optimum parameters of stabilizers are dependent on the formulation preparation method and on the active pharmaceutical ingredients. For these critical properties, researchers make individual evaluations on the basis of active substances in the screening of stabilizers and decide on optimum stabilizers by a simple trial-and-error approach [39].

Some excipients used for the stabilization of NSs, as discussed in several studies, are summarized below (Table 2).

Stabilization in NSs can be steric and/or electrostatic. In steric stabilization, a steric barrier is created by the adsorption of a polymer on the particle surface of the nanocrystals, and aggregation of particles is prevented. In electrostatic stabilization, which is the other mechanism, the NS is stabilized by reducing the surface tension at the interphase interface thanks to electrostatic repulsion from the ionic surfactants added to the particle surface [24]. Stabilizers commonly used in NSs are surfactants such as poloxamer 188, poloxamer 407, vitamin E TPGS, polysorbate 80, sodium lauryl sulfate and polymeric substances such as polyvinyl pyrrolidone, polyvinyl alcohol, and cellulose derivatives (hydroxypropyl methyl cellulose—HPMC, hydroxypropyl cellulose—HPC, hydroxyethyl cellulose—HEC, and Methyl cellulose—MC, etc.) [39].

In NS formulation, the drug:stabilizer ratio (*w/w*) can vary widely, from 1:20 to 20:1 [39,47]. Stabilizers can be used in NS formulations using different preparation methods after the optimum ratio is determined by preliminary studies. In the use of stabilizers, specific choices are not made for drug administration routes. The stabilizers and results obtained in NSs prepared using different preparation methods for different routes of administration are summarized in the following sections of this review.

## 4. Characterization Studies for Nanosuspensions

Physical, chemical, physicochemical, and biological tests are performed for the characterization of the prepared NSs before and/or after solidification. Mean particle size and particle size distribution (polydispersity index), crystalline state and particle morphology, surface charge, saturation solubility, dissolution rate, stability, and in vivo biological performance studies are some of the basic characterizations for NSs. Various characterization methods of nanocrystalline formulations are summarized in Table 3.

## 5. Solidification of Nanosuspension and Stability

Despite the individual and even combined use of the many stabilizers shown in Table 2, it is impossible to completely inhibit the crystal growth of nanosized particles. Thermodynamically, the presence of NSs in a liquid dispersion medium accelerates crystal growth. Therefore, to obtain long-term stability and avoid aggregation, hydrolysis, and other stability problems, the use of appropriate stabilizers, as well as the drying of the formulation, is necessary. The drying process in NSs is undertaken by freeze-drying or spray-drying methods. These dried nanocrystals thus obtained can be presented to patients in solid dosage forms such as powder, tablet, or capsule [40].

To solidify the liquid suspensions, drying methods such as either lyophilization or spray drying are preferred. In cases where the active substance is likely to be affected by heat, the drying process is by lyophilization; the drying method may be selected by spray drying with effective substances that are not affected by heat and in cases where the drying particles should be more spherical.

Freeze drying, the lyophilization method, is the most common method of drying NSs. After a sudden freezing step, primary and secondary drying is performed under a vacuum. In this method, the segregation of nanocrystals as unfrozen small liquid packages and particle aggregation are prevented by using cryoprotectant material. Water-soluble matrix-forming sugars such as mannitol, sucrose, glucose, dextran, and trehalose are used for cryoprotectant purposes [49,58,60].

In the spray-drying method, temperature and pressure are rapidly applied to the NS formulation [47]. The dried particles are spherical, and the flow properties are quite good.

## 6. Administration Routes of Nanosuspensions

NSs can be administered by dermal, parenteral, ocular, and pulmonary routes, as well as by the oral route, which is the most common route for NS administration (constituting more than 60%) [19,49].

### 6.1. Oral Administration

The oral route is the preferred route of administration of drugs in terms of patient compliance, non-invasiveness, ease of use, dose flexibility, and safety. Beside patient benefits, oral dosage forms have advantages regarding cost-effectiveness, feasibility, and suitability for large-scale production. Due to these advantages, it is estimated that 90% of commercial drugs are for oral use [62].

When a drug is given orally, bioavailability and efficacy depend on solubility and absorption in the gastrointestinal tract. Poor aqueous solubility, poor permeability, the effects of being fed or fasted, enzymatic degradation, and the high first-pass effect are challenges that the development of oral medications encounter; they may result in inadequate in vivo absorption and the formulation not reaching an effective therapeutic concentration [63]. In addition, 40–70% of newly discovered drugs emerge with low solubility properties with limited oral bioavailability [64]. Due to the low bioavailability, a drug candidate may have to be administered in larger doses than usual, increasing the cost of treatment [65].

Currently, the Biopharmaceutical Classification System (BCS) is used to identify the physicochemical limitations for oral bioavailability based on the drug solubility properties of the drug throughout the upper gastrointestinal tract. BCS Class II and IV drugs limit both the rate and the extent of drug absorption. The rate of dissolution of the drug is the principal limitation of oral absorption [66]. This is why the dissolution rate can be considered the primary effective parameter for drug pharmacokinetics, which is related to drug solubility and particle size. Increased saturation solubility results in an increased concentration gradient between the gastrointestinal tract and blood and in an increased dissolution rate of the drug. In this way, the adhesiveness of drug particles provides enhanced bioavailability.

Particle size reduction is one of the most common approaches to increasing the saturation solubility and dissolution rate. The micronization process is widely used for this purpose, using colloid mills or jet mills. Micron-sized particles above 1 μm increase the dissolution rate because of the increased surface area. However, this does not change the saturation solubility of a drug, and low oral bioavailability still poses a problem [67]. Although it is known that the saturation solubility of an active substance is a compound-specific property depending on crystalline form, the lipophilicity of the drug, fed/fasted state, pKa, temperature, and properties of dissolution medium, at a nanometer range of saturation solubility, are also functions of particle size, according to the Ostwald–Freundlich and Kelvin equations [16,68,69]. NSs provide a tremendous increase in the surface-area-to-volume ratio and this leads to a higher solubility. Particle size reduction from 10 microns to 200 nm generates a 50-fold increase in the surface-area-to-volume ratio [70]. Higher solubility results in higher Ct and improved dissolution rate. This phenomenon can be explained by the Noyes–Whitney equation [71].

The mechanical properties, surface area, and surface morphology of drug substances affect their properties of adhesion to biological surfaces [72]. With respect to the increased surface area of drugs, NSs form a high concentration gradient between the gastrointestinal tract and blood vessels. A decrease in diffusion layer thickness is provided and this results in the high saturation solubility and dissolution of the drug [73].

In the first studies on the increase in in vivo bioavailability of NSs, the active substance danazol was administered to beagle dogs in nanocrystalline form, and it was determined that oral bioavailability increased approximately 16 times [74]. With the success of NS technology in the research area, various commercial oral products began to appear on the market in 2000. These included Rapamune^®^ (Sirolimus tablets—Company: Wyeth), Tricor^®^ (fenofibrate tablets—Company: Abbott), Focalin^®^ XR (dexmethylphenidate HCl capsules—Company: Novartis), Emend^®^ (aprepitant capsules—Company: Merck), Zanaflex^®^ (tizanidine HCl capsules—Company: Acorda) and Megace^®^ ES (megestrol acetate oral suspension—Company: PAR Pharmaceutical) [75]. The reasons for the preparation of NSs for oral administration and the main problems associated with it are presented in Table 4 [76,77,78].

NSs are obtained as nanocrystal drug particles in an aqueous medium with surfactants or polymers as stabilizers. It is possible to use NSs in liquid dosage form for oral use. Thus, the general advantages of liquid dosage forms such as higher flexibility of dosing, rapid absorption, higher bioavailability, and suitability for patients who suffer from swallowing difficulties can be achieved. While conventional oral suspensions have some excipients as suspending agents or carriers, drug NS systems do not contain any carriers, and they are prepared completely with the parent drug [26]. As a disadvantage, however, NS formulations in liquid have lower physical and chemical stability.

After solidification, NSs can be used as oral powders. Oral powders have better long-term physical and chemical stability. It is also possible to easily wet and redisperse the powders into a suitable liquid. Pellets can be obtained from dry powders by extrusion–spheronization or directly from liquid NSs by the fluid-bed coating method [14]. The pellets can reduce variations in gastric emptying rates and, hence, reduce the intra and inter-subject variability. They disperse freely in the gastrointestinal tract, avoiding high local concentrations that may irritate and drug absorption increases, with minimized potential side effects [79]. In addition, wet granulation or spray drying can be used to obtain granules. Powders and granules can be blended with appropriate excipients and put in capsules or compressed as tablets to achieve patient-compliant dosage forms.

NSs are feasible systems for oral film formulations. Liquid NSs are dispersed in polymer solutions with a plasticizer, and oral films can be obtained by the conventional casting method. The NS-loaded oral film formulations offer ease of preparation, rapid disintegration, no need for water intake, easy administration in the mouth or under the tongue, avoiding first-pass metabolism, and enhanced bioavailability [80]. The most recently published studies on oral NS are summarized in Table 5.

### 6.2. Parenteral Administration

The parenteral route of drug administration is a widely used route of drug administration in clinical practice, with the advantage of reduced dosing, approximately 100% bioavailability, rapid onset of action, independence from the gastrointestinal tract, and avoidance of hepatic first-pass metabolism [90].

NSs for parenteral administration are nanometer size and are frequently prepared because of their ease of permeability, high drug loading capacity, and small volume of administration. In addition, the risks of toxicity and allergic reactions are prevented as a result of the low amount of excipients used in these formulations. While developing a new drug delivery system for parenteral delivery, it should be kept in mind that the delivery system should not be phagocytosed by Kupffer cells in the reticuloendothelial system and liver. Therefore, a size range of ≤100 nm is crucial for parenteral NSs [40].

In addition, during the parenteral administration of nanocrystals in vivo, the duration of blood circulation can be increased by surface modification with substances such as PEG in NSs to prevent opsonization.

Some studies on the parenteral administration of NSs are shown in Table 6.

### 6.3. Pulmonary Administration

While the local effect can be achieved through the pulmonary route, the systemic effect can also be achieved because of the large surface area of the lung, thin alveolar epithelium, and low enzymatic activity [94]. At the same time, the anatomical structure of the respiratory tract provides an appropriate site for the immune response. Particle sizes, shapes, densities, and loads of inhaled drug particles are the leading factors affecting the retention (deposition) of aerosols in the lungs. In addition, the physicochemical properties of the active substance such as solubility, partition coefficient, permeability, molecular weight, enzymatic stability and formulation form, biophysical parameters, and the tools used affect the bioavailability of the inhaled drug. With recent advances in nanotechnology, there has been an increase in research for the development of new pulmonary drug delivery systems for the treatment of various diseases such as chronic obstructive pulmonary disease and asthma [95,96].

Nanocrystalline technology can significantly increase the bioavailability of poorly soluble drugs by reducing particle size and prolonging lung residence time. It provides a potential formulation development strategy for the delivery of drugs to the lungs [97,98,99]. In addition, nanocrystals—as a free-carrier nanotechnology—have gained increasing interest in the pulmonary administration of poorly soluble drugs because of their improved dissolution rate and saturation solubility, biological properties, and the low toxicity of poorly soluble drugs. Problems seen in conventional pulmonary delivery systems such as rapid drug release, poor residence time, and lack of selectivity can be solved with NSs. Furthermore, NSs increase bioavailability by improving drug diffusion and dissolution rate and preventing unwanted drug accumulation in the mouth and pharynx.

For pulmonary administration, NSs can be nebulized using jet or mesh nebulizers or aerosolized via metered dose inhalers [100] and dry powder inhalers [101]. For pulmonary administration, itraconazole [102,103], budesonide [104,105,106], and fluticasone NSs have been developed [107]. Previous studies have shown that nebulized NSs have acceptable aerodynamic performance and several advantages over conventional micronized drugs, including the ability to shorten nebulization time, improve patient compliance, and promote uniform distribution of drugs in the lungs by rapid diffusion [100,105].

Inhalable aerodynamic properties are an important factor affecting the pulmonary inhalation of drugs. The size distribution of respirable particles is usually expressed by the aerodynamic diameter, which varies with the shape, size, and density of the objects. The aerodynamic diameter of respirable particles determines whether they can accumulate in the lungs. Nevertheless, regardless of the method of aerosol administration, strict control of particle size to within the aerodynamic diameter (dA) range of 1–5 µm is necessary for optimal pulmonary delivery. Particles with dA > 5 µm are mostly deposited on the walls of the upper respiratory tract by inertial impaction, while particles with dA < 1 µm tend to remain airborne in the airways and are exhaled during the normal breathing cycle [108].

In addition, there are studies of pulmonary applications of NSs as nanocrystal-based inhalation systems, aerosol, adhesive microparticles, composite microparticles, and mucus-penetrating nanocrystals [109].

Selected pulmonary-route-administered NS study examples are summarized in Table 7.

### 6.4. Ocular Administration

The eye is the most particular organ of the body, and various drug delivery systems were employed to deliver the drugs into the eye. The design of drug delivery systems for ocular administration has become a challenge in the pharmaceutical field [117]. Ocular drug delivery is needed in the treatment of some diseases such as glaucoma, dry eyes, diabetes retinopathy, proliferative vitreoretinopathy, keratoconus, macular degeneration, conjunctivitis, blepharitis, and uveitis. Systemic application used in the treatment of these diseases might have a limited effect because of blood–aqueous and blood–retinal barriers after ocular administration. These barriers can limit the amount of drug that reaches the extravascular retinal space and the aqueous and vitreous humors of the eye. For this reason, local or ocular application of drugs presents a higher drug concentration to the specific site of the ocular region [118,119]. Thus, the main purpose of ocular drug administration is to enhance the number of drugs reaching the specific ocular site and, thus, to improve the therapeutic effect. Although 90% of the marketed ophthalmic formulations are conventional eye drops, the low bioavailability related to the precorneal loss factors (static and dynamic barriers) became a major limitation for their usage. About 5% of the drug can pass through the cornea and reach the intraocular tissue because of vast and quick precorneal drop loss caused by high tear fluid output or blinking. While some ocular ointments have managed to overcome this problem, they also cause a blurring of vision. Controlled drug delivery systems and nanotechnological drug delivery systems have shown promise in tackling these problems. The ocular application of NS is an especially valuable approach to delivering both highly hydrophobic and hydrophilic drugs across the ocular mucosa. The main mechanism of the increase in ocular bioavailability via NS is the increment of dissolution velocity along with saturation solubility of poorly water-soluble drugs. Moreover, NSs can be prepared with various surfactants, viscosity enhancers, or charge modifiers. There is a possibility of a wide range of NS formulation designs that can gain mucoadhesive properties and the controlled release profile and enhance the retention time, permeation, and tolerability on the ocular site [117,120,121]. NS has a low risk of ocular irritation because of using nanosized particles, and the charge on the surface of NS facilitates their adhesion to the cornea. Based on all these advantages, the NSs can solve major issues such as the low contact time and poor ocular bioavailability related to the drainage of drug solution, tear turnover, and dilution or lacrimation [122]. The advantages of ocular NSs are also given in Table 8. NS has been explored for ocular drug delivery by various researchers, and Table 9 shows the application of various NSs in ocular drug delivery.

### 6.5. Dermal and Transdermal Administration

Dermal drug application has many advantages such as reducing side effects, ensuring drug accumulation in the specific area, controlled administration of the drug to the organism, self-administration of the patient, high patient compliance, and providing a specific effect [130,131]. There are two basic approaches to the dermal application of drugs: transdermal and dermal. While the applied formulation is localized in the dermal layers in the dermal application, it passes through the carrier to the lower layer of the skin and then enters the systemic circulation in the transdermal application [132,133]. Dermal and transdermal drug application has advantages as well as disadvantages. Because of the barrier effect of the stratum corneum layer, it is not possible to administer all drugs by this route. Crossing the stratum corneum barrier is only suitable for low-dose/high-permeability drugs. For an active substance to reach the lower layers of the skin, it needs to be small (molecular weight ≤ 500 Da), lipophilic (log*p* value ≤ 1–3), and compatible [134]. If these conditions are not met, sufficient blood concentration may not be reached because of the skin barrier. Adhesive structures used for transdermal purposes may not be suitable for all skin types. Since drugs and drug formulations may cause skin irritation and sensitivity, this situation should be evaluated in the drug development process. The advantages and challenges of dermal/transdermal NSs are presented in Table 10.

In recent years, many nanotechnological systems have been investigated to enhance the effectiveness of drugs after dermal/transdermal application. NSs are an especially promising system among the nanosystems for the dermal/transdermal application of drugs. By decreasing the particle size of the active substance to nanosize with NS technology, increasing the surface area and solubility, and thus the bioavailability, provides superiority in terms of dermal use. With the increase in saturation solubility, the concentration of the active substance on the skin surface increases, and depending on the increase in the concentration gradient, the passage of the active substance through the skin by passive diffusion accelerates [12,14]. In addition, with the increase in the surface area, the spreadability and adhesion of the particles to the skin surface also increase. By choosing positively charged polymers in the structure of NSs, penetration into the negatively charged stratum corneum layer can be increased. Therefore, in recent years, the development of NS formulations has gained importance in increasing the dermal bioavailability of active substances with low or medium water solubility. In 2007, NS formulations of low-soluble antioxidant-effective rutin and hesperetin-active ingredients have been developed, and the first effective NS-based anti-aging cosmetic preparation has been introduced to the market [135,136].

Table 11 shows NS formulations developed for dermal use.

When NSs are applied dermally, they can have a local effect by penetrating the skin surface, or they can have a systemic effect by passing under the skin through intercellular hydrophilic routes, depending on the increase in saturation solubility. However, it is thought that the depot effect formed by the accumulation of particles in the hair follicles is more effective in the passage of NSs through the skin [145]. Carrier systems such as creams, anhydrous ointments, or gels are used to facilitate the dermal application of NSs and to increase their effectiveness. Thus, the residence time on the skin surface can be extended or the release of the active substance can be controlled depending on changes in viscosity. On the basis of all these advantages and findings in new studies, NSs are now understood to be a very promising system for dermal application.

## 7. Challenges and Future Perspective

Many newly discovered drug molecules are in BCS Class II and have very low water solubility. With the increasing number of these low-soluble drugs, which are not able to be formulated via traditional approaches, NSs have recently gained more importance. The advantages of NSs, such as applicability to a broad range of drugs, ease of scale-up, minimum use of excipients, and increased solubility followed by increased dissolution rate and bioavailability, lead to their broad acceptance in the development of formulations. NSs that allow drug administration by the dermal, pulmonary, parenteral, and ocular routes, especially the oral route, can be used successfully in various diseases for therapeutic purposes. These advantages are mainly reflected in the increasing number of NS-based commercial products. In addition to existing commercial products, further commercialization of NSs is likely with the future conclusion of clinical studies into various administration routes. At present, even though the NS formulations have progressed significantly, there are limited in vivo studies and clinical trials and also many problems in the selection of stabilizers, maintenance of stability, and other aspects. There is, therefore, a need to increase the number of clinical trials, to enrich the pharmacokinetic data after the administration of various NSs, and to establish theoretical models to identify the formulation development and optimization process of NSs. Moreover, some supporting equipment and technologies that are additionally providing high stability and providing easy scaling up will be more important in the future. The value of the technology and principle of NS formulations can be assessed by considering the number of products in clinical phases and in the market, paying attention also to the dates of entry into the market.

## Figures and Tables

**Table 1:** Comparison of advantages and disadvantages of nanosuspension preparation methods.

Preparation Method	Advantages	Limitations
Bottom-up technology	Simple in principle and operation No device requirement Rapid preparation	Poor reproducibility Non-homogeneous particle size Risk of toxic effects of the solvent Difficult to scale up
Top-down technologies		
High-pressure homogenization method	Easy to scale up Easy to reproduce Obtaining homogeneous particle size Obtaining the desired particle size with process modifications Decreasing for recrystallization	Expensive equipment Risk of heating in the device because of high pressure Risk of clogging the chamber
Wet media milling method	Easy to scale up Easy to reproduce Obtaining homogeneous particle size Obtaining the desired particle size with process modifications	Expensive equipment Risk of corrosion of beads and milling chamber

**Table 2:** Stabilizers used in nanosuspension formulations.

Stabilizer	Stabilizer Type	Structure	References
Cellulose derivatives	Polymeric stabilizer	A cellulose derivative of cotton natural or synthetic fibers	[35,42,43]
Polyvinyl alcohol (PVA)	Polymeric stabilizer	A synthetic water-soluble resin obtained from the hydrolysis of polyvinyl acetate	[44,45,46]
Polyvinyl pyrrolidone (PVP)	Polymeric stabilizer	A synthetic linear-chain water-soluble polymer fabricated from the polymerization of the monomer N-vinylpyrrolidone	[32,47,48]
Polyethylene glycols	Polymeric stabilizer	A hydrophilic polymer of ethylene oxide	[38,42,49]
Sodium lauryl sulfate (SLS)	Surfactant	A sulphuric acid mono-dodecyl ester sodium salt	[36,47,48]
Plantacare^®^ 2000	Surfactant	A plant-derived feedstock	[12,29,50]
Brij derivatives	Surfactant	A polyoxyethylene alkyl ether	[51,52,53]
Lecithin	Surfactant	A mixture of phosphatides with triglycerides, fatty acids, and carbohydrates	[49]
D-α-tocopheryl polyethylene glycol 1000 succinate (vitamin E TPGS/ TPGS 1000)	Surfactant	An ester of vitamin E with PEG 1000	[32,51]
Poloxamers	Surfactant	Amphiphilic block copolymers	[54,55,56]
Polysorbate 80	Surfactant	A polyoxyethylene sorbitan fatty acid ester derivative	[41,57]

**Table 3:** Characterization studies for nanosuspensions.

Characterization	Methods	Principle	Significance	References
Particle size and morphological evaluation	Dynamic light scattering (DLS) and photon correlation spectroscopy (PCS)	Fluctuation of Rayleigh scattering of light associated with Brownian motion of nanoparticles	Particle size (PS) and particle size distribution (PDI) measurements	[19,49]
	Optical microscopy, scanning electron microscopy (SEM), transmission electron microscopy (TEM), and stomic force microscopy (AFM)	Reflection or transmission of electrons incident on the particle and the force applied to the sample by the probe	Particle size measurement, surface morphology, and three-dimensional image	[24,48,58]
Surface properties	Dynamic light scattering (DLS)	Electrophoretic mobility	Surface charge (zeta potential—ZP) measurements	[40]
Solid state (Structural) characterization	Differential scanning calorimetry (DSC) and differential thermal analysis (DTA)	Thermogravimetric analysis and physical change in the sample versus change in heat flow	Solid state form analysis (enthalpy, melting point, glass transition temperature)	[58]
	Infrared (IR) spectroscopy (mid-IR and Fourier-transformed IR spectroscopy) and Raman Spectroscopy	Change in dipole moment during molecular vibrations and in polarizability during molecular vibrations	Polymorphic form changes (analysis of amorphous, crystalline, and polymorphs)	[59]
	X-ray powder diffraction (XRPD)	Diffraction of X-rays transmitted on the sample	Polymorphic form changes (analysis of amorphous, crystalline, and polymorphs)	[60]
Rheological properties (for liquid nanosuspensions)	Viscometer and rheometer	The way a liquid flows in response to the applied force and the viscosity of a fluid	Rheological character and flow type	[48,61]
Solubility	Ultraviolet (UV) spectrophotometer and high-performance liquid spectroscopy (HPLC)	Detection of increase in saturation solubility using spectroscopy or chromatography	Increasing active substance solubility	[49,60]

**Table 4:** Features of nanosuspensions in oral drug delivery.

Reasons for the Development of Oral Nanosuspensions	Challenges to Be Overcome	Specific Studies
Enhancement of solubility and dissolution Improved bioavailability Improved absorption Providing better drug stability Dose proportionally The rapid formulation development process Eliminating fed/fasted variability Reduced inter-subject variability Using directly as liquid dosage form Convertibility to solid dosage form (tablets, capsules, pellets, oral films, powders, granules) Ease of large-scale production Increasing mucoadhesive and attachment to cell membranes Reduction in drug dose	Challenging in selection stabilizer type and ratio Overcoming physical (Ostwald ripening, agglomeration) and chemical (hydrolysis) stability issues Usually requires solidification	Saturation solubility Dissolution rate Stability In vivo biological performance (pharmacokinetic and pharmacodynamic)

**Table 5:** Recent studies on the oral administration of nanosuspensions.

Drug	Use/Treatment	Stabilizers	Preparation Method	Characterization	Outcomes	References
Gliczaide	Antidiabetic	SDS, Lecithin	Solvent–Antisolvent Precipitation	PS: 96.49 ± 15 nm PDI: 0.326 ± 0.05 ZP: −22 ± 5.6 mV	The C_max_ and AUC_0–t_ values of NS were approximately 3.35- and 1.9-fold higher than those of the raw medication and marketed formulation.	[81]
Silymarin	Hepatoprotective	PVA	Solvent–Antisolvent Precipitation	PS: 277.3 ± 10.4 nm PDI: 0.114 ± 0.075 ZP: −22.8 ± 2.8 mV	Saturation solubility of nanosuspensions enhanced 3.48 times compared to the coarse powder, improved dissolution.	[46]
Ziprasidone	Antipsychotic	PVP K30	Microfluidization	PS: 600 nm PDI: 0.4 ZP: 29 mV	The solubility of nanosuspensions was increased up to 2.3-fold compared with the coarse powder. Nanosuspensions showed >95% dissolution in the FeSSIF medium and 80% in the FaSSIF medium.	[32,82]
Cyclosporine A (CsA)	Immunosuppressive	HPMC, SDS	Wet milling	PS: 600 nm PDI: 0.4 ZP: −25 mV	The solubility of CsA was increased 4.5-fold by nanosuspensions. AUC_0–24_ values of CsA nanosuspension were to be 2.09 and 5.51-fold higher than coarse powder in fasted and fed conditions. C_max_ was 3.99-fold higher than coarse powder.	[36,43]
Ritonavir (RTV)	Antiprotease HIV	HPMC, SDS	Microfluidization	PS: 540–550 nm PDI: 0.1–0.4 ZP: −20 mV	The solubility of nanosuspension was enhanced five times. 57% and 18% of RTV were dissolved in FeSSIF medium for nanosuspension and coarse powder. C_max_ and AUC_0−t_ values in nanosuspension displayed an 8.9- and a 12.5-fold increase, respectively, compared to the coarse powder, and a 1.9- and 2.1-fold increase, respectively, compared to the commercial product.	[11,83]
Paroxetine	Depression and anxiety	Poloxamer 188	Solvent–Antisolvent Precipitation	PS: 217.09 ± 4.18 nm PDI: 0.46 ± 0.27 ZP: −33.49 ± 2.08 mV	Increase in C_max_ (1.74-fold), AUC_0–48_ (1.56-fold), and AUC_0–∞_ (1.78-fold), when compared with the market tablet.	[84]
Canagliflozin	Type 2 diabetes mellitus	Poloxamer 407	Wet milling	PS: 120.5 ± 5.6 nm PDI: 0.217 ± 0.23 ZP: −23.0 ± 4.75 mV	Pellets released more than 89% drug within 10 min as compared to the marketed tablet and pure drug, which released 24.63% and 18.65% of the drug, respectively, within 10 min.	[85]
Lumefantrine	Anti-malarial	Polysorbate 80	Anti-solvent precipitation and ultrasonication	PS: 168.3 nm PDI: 0.128 ZP: −25.7 mV	Saturation solubility increased in nanosuspension (1670 mg/mL) when compared to the pure drug (212.33 mg/mL). Lyophilized nanosuspension showed an 8-fold increase in drug release.	[86]
Indomethacin	Anti-inflammatory	PVP, SDS	Wet milling	PS: 195 ± 7 nm PDI: 0.12 ± 0.02	Coarse powder released 49 ± 2% after 60 min while nanosuspensions released >95% after 30 min.	[87]
Doxazosin Mesylate	Antihypertensive	PVP K 30, Poloxamer 407, SLS	Emulsification solvent diffusion	PS: 385 ± 13.00 nm PDI: 0.049 ± 3.33 ZP: 50.33 ± 4.20 mV	Significant reduction in mean arterial blood pressure of hypertensive rats for more than 3 h when compared with marketed tablet; 100% dissolution after 10 min.	[88]
Curcumin	Anti-inflammatory, antiviral, antibacterial, and antitumor	SDS, PVP/PVA	Anti-solvent precipitation	PS: 127.7–1.3 nm PDI: 0.227–0.010	More than 80% of the drug is released. The maximum drug plasma concentration of the tannic acid-coated nanosuspension formulation was 7.2-fold higher than that of the pure drug.	[89]

PS: particle size, PDI: particle size distribution, ZP: zeta potential, NS: nanosuspension, SDS: sodium dodecyl sulfate, PVA: polyvinyl alcohol, PVP: polyvinyl pyrolidone, FeSSIF: fed-state simulated intestinal fluid, FaSSIF: fasted-state simulated intestinal fluid, HPMC: hydroxypropyl methylcellulose, SLS: sodium lauryl sulfate.

**Table 6:** Some studies on the parenteral administration of nanosuspensions.

Drug	Use/Treatment	Stabilizers	Preparation Method	Characterization	Outcomes	References
Asulacrine	Anticancer	Poloxamer 188	High-pressure homogenization	PS: 133 ± 20 nm	Enhanced solubility (app. 40-fold). Reduced C_max_ and AUC_0–∞_ and greater AUC_0–∞_ in liver, lung, and kidney compared to solution.	[91]
Curcumin	Anticancer	Cremophor EL-40, Tween 80, Poloxamer 188, SDS, HPMC, Carbomer 940	Nanoprecipitation, High-speed homogenization, High-pressure homogenization, Combined nanoprecipitation and high-pressure homogenization	Best suspending effect with soya lecithin Successfully prepared by high-pressure homogenization PS: 250.6 nm ZP: −27.92 mV	Solubility and dissolution rates were significantly increased. Superior cytotoxicity in Hela and MCF-7 cells. Less local irritation and phlebitis risks, lower rate of erythrocyte hemolysis.	[92]
Bexarotene	Anticancer	Poloxamer 188, Soybean lecithin, PVP K30	Precipitation-combined microfluidization method	PS: 279.0 ± 3.2 nm PDI: 0.104 ± 0.014	Improved solubility (app. 10-fold). Higher AUC, C_max,_ and a longer mean retention time.	[93]
p-terphenyl derivative (H2)	Anticancer	Poloxamer 188, Lecithin	Combined microfluidization and precipitation method	PS: 201.7 ± 5.87 nm ZP: −21.07 ± 0.57 mV	Increased saturation solubility and accelerated dissolution velocity. 5-fold higher AUC_0∼∞_. A longer mean retention time.	[60]

**Table 7:** Example studies on the pulmonary administration of nanosuspensions.

Drug	Use/Treatment	Stabilizers	Preparation Method	Characterization	Outcomes	References
Budesonide	Asthma	HPMC, SLS	Microfluidization	PS: 122.5 ± 6.3 nm ZP: 13.6 ± 0.4 mV	The dispersion of the nanosuspensions in the lung was easier than normal particles and micronized particles. After 1 h of inhalation, the drug concentration reached 872.9 ng/g. This differs significantly from normal particles (*p* < 0.01) and micronized particles (*p* < 0.05).	[110]
Budesonide	Asthma	Lecithin, Span 85, Tyloxapol	Homogenization	Formulation (contain lecithin) PS: 599 nm PDI: 0.278 ZP: −12 mV Formulation (contain Tyloxapol) PS: 500 nm PDI: 0.397 ZP: −41.1 mV	The results showed that a long-term stable pulmonary budesonide nanosuspension could be used with a conventional nebulizer or with a portable inhaler system.	[104]
Curcumin (CUR) and Beclomethasone Dipropionate (BDP)	Bronchial asthma	Poloxamer 188	Wet ball media milling	CUR-NS PS: 202 nm PDI: 0.25 ZP: −30 mV CUR+BDP-NS PS: 240 nm PDI: 0.24	Improved CUR apparent solubility by approximately, 54-fold comparison with the raw material. The results suggest that the formulation should be delivered accurately and efficiently to deeper lung regions, showing multicomponent nanosuspension, optimal dimensional properties, and aerodynamic parameters.	[111]
Fluticasone propionate (FP)	Corticosteroid	EDTA-2Na, NaCl, Sodium citrate, Citric acid, Tween 80	Combined wet milling with high-pressure homogenization	PS: 246 ± 2.94 nm PDI: 0.20 ± 0.04 ZP: 0.35 ± 0.14 mV	This study demonstrated that inhalable nanosuspensions are a viable vehicle for sustained pulmonary delivery of FP and their local anti-inflammatory activity is largely dependent on their dissolution profile. Intratracheally dosed nanosuspensions attenuated mucociliary clearance and prolonged pulmonary absorption time and improved local retention, resulting in a significant prolongation of the local anti-inflammatory effect of FP.	[112]
Loratidine	Allergic rhinitis, urticaria, and atopic dermatitis	Stabilizer mixtures of Tween 80 or Pluronic F68 + PVP-K25	Ultrasonic-assisted precipitation	PS: 353–441 nm PDI: 0.167–0.229 ZP: −25.7–−20.7 mV	This study demonstrates that preparing dried loratadine nanoparticles suitable for designing effective drug preparations is a feasible approach.	[113]
Itrocanozole (ITRA)	Allergic Bronchopulmonary Aspergillosis (ABPA) Cystic fibrosis (CF)	Poloxamer 188, Polysorbate 80, Solutol H15	Wet milling method	Solutol HS 15 formulation: 300 nm Formulation using polysorbate 80: 180–210 nm PDI: low for both polysorbate 80 and Solutol	The results indicate that ITRA nanosuspension represents an interesting formulation for inhaled administration in CF patients suffering from ABPA. High and long-lasting lung tissue concentrations well above the minimal inhibitory concentration of Aspergillus species enable once-daily administration with minimal systemic exposure.	[114]
Mometasone Furoate Monohydrate (MFM) combined with Formoterol Fumarate Dihydrate (FFD)	Asthma	DPPC	High-pressure homogenization and spray-drying process	Aerodynamic diameter MFM: 1.71 ± 0.04 µm FFD: 2.20 ± 0.44 µm	The results clearly showed that the combination of homogenization and spray drying methods is suitable to obtain DPI formulation containing MFM and FFD with particle size less than 5 µm to reach alveoli.	[115]
Telmisartan	COVID-19 Lung Disease and Other Respiratory Infections	Polysorbate 80	Probe sonication	Hydrodynamic diameter PS: 322 ± 15 nm PDI: 0.24 ± 0.03 ZP: −2.9 ± 0.5 mV	The developed nanosuspension demonstrated excellent applicability to the lungs, pharmacokinetics, and acceptable tolerability in rodents and/or non-human primates. Clinical evaluation of the formulation for inhaler use in patients with COVID-19 or other respiratory diseases is ongoing.	[116]

PS: particle size, PDI: particle size distribution, ZP: zeta potential, HPMC: hydroxypropyl methylcellulose, SLS: sodium lauryl sulfate, EDTA: ethylenediaminetetraacetic acid, PVP: polyvinyl pyrolidone, DPPC: dipalmitoylphosphatidylcholine, COVID: coronavirus disease.

**Table 8:** Features of nanosuspensions in ocular drug delivery.

Reasons for the Development of Ocular Nanosuspensions	Challenges to Be Overcome	Specific Studies
Increased saturation solubility and dissolution Increased permeation İncreased contact/retention time on the ocular site Increased transcorneal penetration Enhancement of specific effects on the ocular site Enhancement of ocular bioavailability Providing better drug stability The rapid formulation development process Using directly as liquid dosage form Convertibility to semi-solid dosage form (cream, ointment, gel, etc.) Possibility for controlled released systems Possibility of mucoadhesion on the ocular site Reduction in drug dose Reduction in side effects Reduction in toxicity	Challenging in selection of stabilizer type and ratio Challenging to design formulation for crossing the cornea and reaching the intraocular tissue Overcoming physical (Ostwald ripening, agglomeration) and chemical (hydrolysis) stability issues Usually requires some excipients such as viscosity enhancers, pH and charge modifiers, or a carrier to apply Requirement for sterilization	pH values Rheological/mechanical properties Surface tension Saturation solubility Stability Corneal residence time In vitro release/ex vivo ocular permeation In vitro transcorneal penetration Ocular irritation In vivo performance after ocular application Corneal bioavailability

**Table 9:** Recent studies on the ocular administration of nanosuspensions.

Drug	Use/Treatment	Stabilizers	Preparation Method	Characterization	Outcomes	References
Hydrocortisone, Prednisolone, Dexamethasone	Conjunctiva	Pluronic F68, EDTA, benzalkonium chloride, hydroxyethyl cellulose	High-pressure homogenization	PS: 650–880 nm	NSs exhibited a higher intensity of drug action and a higher extent of drug absorption.	[123]
Hydrocortisone	Inflammation	PVP, HPMC, Tween 80	Microfluidic nanoprecipitation and wet milling	PS: 295–300 nm PDI: 0.18	The nanosuspensions showed sustained action and enhanced bioavailabilities compared to the hydrocortisone solution, moreover improved stability.	[124]
Triamcinolone acetonide	Inflammation	Poloxamer 407, PVA	Nanoprecipitation technique	PS: ~150 nm PDI: ~0.3	Using the NS, improved loading capacity and solubility, and high physical stability were obtained.	[125]
Acetazolamide	Ocular hypertension	PVA, Soya bean lecithin, HY or PG	Antisolvent precipitation technique + sonication	PS: 100–300 nm ZP > ±20 mV	Enhanced saturation solubility and efficient ocular hypotensive activity were obtained. The modified Draize test showed tolerability and safety on the eye.	[126]
Brinzolamide	Ocular hypertension	HPMC, Pluronic F127 or F68, Polysorbate 80	Wet milling	PS: 460–530 nm PDI: 0.12–0.21	The NSs were homogenous and stable. They dissolved immediately in vitro and provided significantly decreased intraocular pressure values.	[127]
Ciclosporin A	Keratoconjunctivitis	PVA, PVP, HPMC, HPC, HEC	Media milling	PS: ~530 nm	Using nanosuspension (with PVA stabilizer), less irritation to the eye was observed compared to the marketed product Restasis^®^.	[128]
Loteprednol Etabonate (LE)	Inflammation	Pluronic^®^ F127	Media milling	PS: ~200–241 nm PDI < 0.15	An increased level of LE in ocular tissue/fluids and an improved pharmacokinetic profile (3-fold higher C_max_)in the ocular tissues of rabbits were observed compared to Lotemax 0.5% suspension.	[129]

PS: particle size, PDI: particle size distribution, ZP: zeta potential, EDTA: ethylenediaminetetraacetic acid, NS: nanosuspension, PVP: polyvinyl pyrolidone, HPMC: hydroxypropyl methylcellulose, PVA: polyvinyl alcohol, HY: hyaluronic acid, PG: poly-γ-glutamic acid, HPC: hydroxypropyl cellulose, HEC: hydroxyethyl cellulose.

**Table 10:** Features of nanosuspensions in dermal drug delivery.

Reasons for the Development of Dermal/Transdermal Nanosuspensions	Challenges to Be Overcome	Specific Studies
Increased saturation solubility Increased skin penetration Increased permeation Increased follicular accumulation Enhancement of specific effects on the side of the skin Enhancement of dermal bioavailability Availability for local and systemic effect Providing better drug stability The rapid formulation development process Convertibility to semi-solid dosage form (cream, ointment, gel, etc.) Possibility for controlled released systems Reduction in drug dose Reduction in side effects	Challenging in selection of stabilizer type and ratio Challenging to design formulations for crossing the skin barrier Overcoming physical (Ostwald ripening, agglomeration) and chemical (hydrolysis) stability issues Usually requires solidification Usually requires a vesicle or a carrier to apply	pH values Rheological/mechanical properties Saturation solubility Stability In vitro/ex vivo skin permeation In vivo performance after dermal application Skin irritation

**Table 11:** Recent studies on dermal administration of nanosuspensions.

Drug	Use/Treatment	Stabilizers	Preparation Method	Characterization	Outcomes	References
Diclofenac sodium (DCF)	Inflammation	Poloxamer 188	Wet milling	PS ∼ 300 nm PDI ∼ 0.2 ZP ∼ −35 mV	In the application of the NSs having double drug concentration, the accumulated and permeated amount of DCF did not change because of the saturation solubility of DCF being constant.	[137]
Curcumin	Acne	Plantacare^®^ 2000, Plantacare^®^ 1200, Plantacare^®^ 810	Smart Crystal^®^ (Wet milling + HPH)	PS: ∼170–180 nm PDI ∼ 0.2 ZP: −30 mV or above	The drug concentration of NS can be 0.2% (for cost-effective drugs) and 0.02% (for very low soluble drugs). The low viscosity of dermal formulations provides enhanced penetration into the skin and follicular targeting/accumulation.	[138]
Nitrofurazone (NTF)	Antioxidant and anti-inflammatory	HPMC E3, PVP K30, HPMC E5 (alone or in combination with surfactants) Poloxamers 188, SDS, Tween 80, TPGS	Wet milling	PS: ∼300 nm PDI: ∼0.2 Stability index (SI): 0.8	The dissolution of NTF nanogel was higher compared to the NTF marketed gel. The permeated amount of NTF through the skin of nanogel after 24 h was higher than the marketed gel in the ex vivo rat skin permeation studies. After the application of NTF nanogel, the retained amount of NTF in rats’ skin was 5.5 times higher than the NTF marketed gel.	[139]
Rutin	Antifungal	Polysorbate 80, Glycerol, Euxyl^®^ PE 9010	Smart Crystal^®^ (Bead milling + HPH)	PS: 240–282 nm PDI: 0.215	Rutin nanocrystals showed increased skin penetration and increased in vitro antioxidant activity	[136]
Cyclosporin A	Antioxidant	TPGS, Kolliphor TPGS	Wet milling	PS ∼ 350 nm PDI: 0.35	The improved skin penetration with higher stable, formulations were successfully obtained.	[140]
Glabridin (GLB)	Psoriasis	Poloxamer 188, PVP K30	Nanoedge^TM^ (anti-solvent precipitation-homogenization)	PS ∼ 149.2 nm PDI: 0.254	Compared to the coarse suspension and physical mixture, NS enhanced the drug permeation flux of GLB through rat skin with no lag phase both in vitro and in vivo. The GLB-NS did not show any significant aggregates and showed a GLB loss of 5.46% after storage for three months at room temperature.	[141]
Flurbiprofen (FB)	Analgesic and anti-inflammatory	Plantacare^®^ 2000 UP (PL)	HPH	PS: 665 nm–700 nm PDI: 0.2–0.3 ZP ∼ −30 mV	The saturation solubility of FB was increased 5.3-fold with NS. The permeability of FB NS was higher than the FB solution in rat skin. The DoE approach was a useful tool for the preparation of FB-NS.	[12]
Flurbiprofen (FB)	Analgesic and anti-inflammatory	Plantacare^®^ 2000 UP (PL)	Wet milling	PS: 237.7 ± 6.8 nm PDI: 0.133 ± 0.030 ZP: −30.4 ± 0.7 mV	In the pharmacokinetic studies, NS gel showed higher permeation and enhanced plasma-blood concentration of FB in rats compared to gels containing coarse suspension and physical mixture.	[142]
Flurbiprofen (FB)	Analgesic and anti-inflammatory	Plantacare^®^ 2000 UP (PL)	Wet Milling	PS: 237.7 ± 6.8 nm PDI: 0.133 ± 0.030 ZP: −30.4 ± 0.7 mV	According to characterization studies of the various gels containing NS, the HPMC gel was found better than others. The anti-inflammatory and analgesic activities of FB were increased by the FB-NS-based HPMC gel compared to the physical mixture-based and the FB coarse powder-based gels.	[143]
Flurbiprofen (FB)	Analgesic and anti-inflammatory	HPMC, PVP K30, Plantacare^®^ 2000 UP, Tween 80	HPH	PS: 593–805 nm PDI: 0.15–1 ZP: −18.5–−38.6 mV	PL stabilized FB-NS protected the crystalline state. The PL is a more efficient stabilizer to obtain smaller PS and more stable NSs. The PL and PVP provided better morphology than others.	[29]
Ibuprofen (IBU)	Anti-inflammatory	Vitamin E TPGS, HPMC K4	Wet milling	PS: 284.5–854.6 nm PDI: 0.211–0.502	A clear correlation was determined between the vitamin E TPGS and particle size of nanocrystals with the flux of IBU through the skin.	[144]
Etodolac (ETD)	Analgesic and anti-inflammatory	PVP K30	Wet milling	PS: 188.5 ± 1.6 nm PDI: 0.161 ± 0.049 ZP: −14.8 ± 0.3 mV	In vitro and ex vivo permeation studies showed that NS-based HPMC or HEC gels were better in terms of enhancing the penetration of ETD because of increased saturation solubility. The enhanced anti-inflammatory and analgesic activity of NS-HEC gels was observed compared to the control and physical mixture.	[35]

PS: particle size, PDI: particle size distribution, ZP: zeta potential, NS: nanosuspension, HPH: high-pressure homogenization, HPMC: hydroxypropyl methylcellulose, PVP: polyvinyl pyrolidone, SDS: sodium dodecyl sulfate, TPGS: vitamin E polyethylene glycol succinate, DoE: design of experiment, HEC: hydroxyethyl cellulose.

## Data Availability

Not applicable.
